# Hybrid Network Defense Model Based on Fuzzy Evaluation

**DOI:** 10.1155/2014/178937

**Published:** 2014-01-16

**Authors:** Ying-Chiang Cho, Jen-Yi Pan

**Affiliations:** Department of Electrical Engineering, National Chung Cheng University, Chiayi 62102, Taiwan

## Abstract

With sustained and rapid developments in the field of information technology, the issue of network security has become increasingly prominent. The theme of this study is network data security, with the test subject being a classified and sensitive network laboratory that belongs to the academic network. The analysis is based on the deficiencies and potential risks of the network's existing defense technology, characteristics of cyber attacks, and network security technologies. Subsequently, a distributed network security architecture using the technology of an intrusion prevention system is designed and implemented. In this paper, first, the overall design approach is presented. This design is used as the basis to establish a network defense model, an improvement over the traditional single-technology model that addresses the latter's inadequacies. Next, a distributed network security architecture is implemented, comprising a hybrid firewall, intrusion detection, virtual honeynet projects, and connectivity and interactivity between these three components. Finally, the proposed security system is tested. A statistical analysis of the test results verifies the feasibility and reliability of the proposed architecture. The findings of this study will potentially provide new ideas and stimuli for future designs of network security architecture.

## 1. Introduction 

Network and information security is a critical link in a country's overall national security system. Once a war breaks out, the network will become a part of the battlefield. When a cyber war starts, no one—from governments to private enterprises—will be spared, similar to a war in real life [1, 2]. Cyber terrorism may not cause human casualties or fatalities, but the amount of damages that it can bring will definitely result in a wider scope. The disaster that it will cause is also likely to be a more devastating one [3, 4]. Hence, the security of networks and information systems is as important as military security. Along with the growing popularity of the Internet, the importance of network security has become increasingly prominent. Users' requirements and expectations of network security have also become more sophisticated, leading to the development and growth of network security technologies.

## 2. Network Defense Technologies

Firewalls can be used for segregating networks with different levels of security requirements. They also have the ability to control the interactions (if any) between various networks. A firewall is essentially a two-way mechanism of security management: it blocks external intrusions and limits outgoing communications by the internal host [5, 6]. In particular, it allows only certain designated information to pass through, irrespective of whether this information is external information trying to enter the intranet or that being transmitted by the intranet to the outside world. In either case, the process cannot proceed until the authentication procedures of the firewall have been carried out by the host. These procedures are carried out on the basis of the settings implemented in accordance with an organization's security rules and policies. Depending on their evaluation of the degree of risk that the network is facing, system administrators have the flexibility to adjust the security controls accordingly [7, 8].

An intrusion detection system (IDS) [9, 10] can be set up to facilitate a network's resistance against external attacks. The IDS collects information on its own, as well as from other systems and the Internet. It then matches the collected information against its database of attack signatures, analyzes potential attack scenarios, and takes the corresponding actions. The main functions of the IDS include the following: (i) identification of the intruder(s); (ii) identification of the intrusion(s); (iii) monitoring and surveillance of security breaches; and (iv) provision of important information in a timely manner during the fight against an intrusion, so as to prevent its occurrence or escalation [11–13].

A honeynet works like an intelligence collection system, whereas a honeypot is deliberately set up as a target to lure hackers. After an intrusion, it will provide an analysis of the hacker's modus operandi, keep one abreast of the latest attack on the server, and highlight the existing network vulnerabilities. One of its other functions is to tap the communications between hackers in order to compile a list of the various tools that they employ [14–16]. A virtual computer network operates from the server of a virtual computer system that runs VMware or User-Mode Linux. A virtual system can comprise several virtual computers operating from a single host system. A virtual honeynet helps to reduce costs, machinery space, and the difficulty of managing a honeypot(s). Further, a virtual system typically allows the “freeze” and “resume” functions, which terminate operations when the computer is being threated; thus, one can analyze the mode of attack and take the necessary action(s) to deal with the situation [17, 18].

The core design concept for an intrusion prevention system (IPS) [19] revolves around immediate detection and active defense. In order to realize this concept, many technological breakthroughs have been achieved by IPS, leading to advantages that cannot be underestimated [20–22]. These can be elaborated as follows.

- In-line installation: The technologies and functions of instant detection under an IDS have been retained, with the additional feature of firewall-protected online installation (i.e., data are directly embedded in the network traffic). Incoming data packets from external systems are passed through a network port. After being checked to ensure the absence of unusual activities or suspicious contents, the data packets are then passed through another port before being sent to the intranet.
- Real-time interdiction: An IPS has powerful, real-time blocking capabilities, allowing it to take preemptive action to block intrusions and intercept intrusive network traffic, thereby avoiding potential damages.
- Advanced detection technology: All data packets that pass through an IPS must be preprocessed and restructured by the hardware to authenticate their specific application protocols [23, 24]. Next, the restructured data packets are screened by the IPS and matched against the identifying features and attack modes of various application protocols. Suspicious data packets are sent to a specialized signature library for comparison, thus improving the quality and efficiency of detection.
- Built-in special rule: An IPS allows the implementation of special rules to block malicious programs from running. It can also assist in the implementation of an acceptable use policy (AUP), including the prohibition of the use of applications that consume large amounts of bandwidth, such as peer-to-peer file sharing and free Voice over Internet Protocol (VoIP) phone calls [22, 25].
- Self-study and self-adaptation abilities: An IPS must have the self-study and self-adaptation capabilities of artificial intelligence to respond to the myriad forms of attacks by hackers. It has to analyze and extract new attack signatures on the basis of the network's communication environment and intrusion statuses, before updating its signature library and formulating a new security and defense strategy [22, 26, 27].

## 3. Network Defense Model

The test subject for our study is a classified and sensitive network laboratory that belongs to the academic network. Its network security model was developed from scratch and gradually built into a complete system. The attempt was to establish a truly effective network defense method. The related procedure is as follows.

- Design a hybrid firewall module. This serves to segregate the intranet from the main gateway to the external network and imposes strict control over access to the intranet resources by external users.
- Install an IDS at a critical node of the network (e.g., the server farms). The purpose of this step is to conduct real-time monitoring and detection of a variety of network activities and create appropriate records and issue early warnings when attacks occur.
- Adopt the honeynet technology to link up the network's hybrid firewall, IDS, and virtual honeynet, and then enable the three components to interact with one another. This creates an early warning system for network security. The system administrators will be promptly alerted when there are intrusions or system vulnerabilities; thus, timely repairs and maintenance can be carried out.

**Figure 1:**
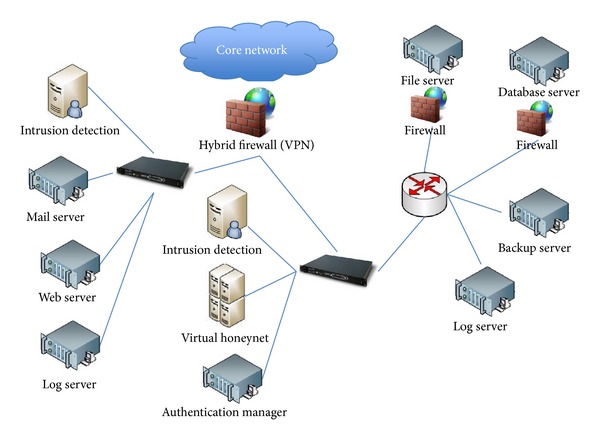
Network defense model.

The topology of the network defense model adopted in this study is shown in Figure 1.

## 4. Research Method and Theory

The module for screening data packets consists of the screening program (which can be operated at the gateway to the firewall) and two backend programs. This module functions as the security router between the network and the data link layers. It intercepts data packets that pass through the gateway of the firewall and verifies whether these contain application protocols that match the preinstalled security regulations, before deciding whether to forward, block, or discard the packets.

The firewall system uses the Netfilter/Iptables framework [28, 29] of the Linux environment. Appropriate modifications were made so that the system meets the requirements of connectivity and interactivity between the internal components of the computer. In this study, we used Iptables, packets selection tool based on the Netfilter framework, to develop a firewall subsystem with various functions. These Iptables include network address translation (dynamic NAT) during the screening of data packets, proxy servers, and others.

Further, the implementation of the firewall system under the Linux environment consists of two aspects. First, Netfilter provides a scalable and structured underlying framework, on top of which Iptables are implemented. The latter is a selection tool, responsible for the filtering and management of incoming and outgoing data packets. Second, Netfilter and Iptables jointly form the main Linux firewall system [30–32].

### 4.1. Interception and Filtering of Data Packets

The program flow for this process is shown in Algorithm 1.

### 4.2. Dynamic NAT

Its role is to conceal the Internet Protocol (IP) address of the internal host so that the security of system can be improved further. A Linux IP masquerading technology was used, making it necessary for the firewall to maintain a dynamic mapping table and update it all the time.

Files related to the masquerading function are included in Algorithm 2.

The standard headers include Algorithm 3.

Among the aforementioned files, the most important is ip_masq.c. It defines the interface of the application layer and the actual process of masquerading the IP address. The other files are extensions of the application.

### 4.3. Fuzzy Representation of the IDS Features

The fuzzy theory is suited for use in intrusion detection because it can easily combine input data from various sources. Since many types of intrusions cannot be clearly defined [33–35], the advance warnings that they trigger are usually vague too.

Fuzzy mathematics is used for describing, researching, and managing the mathematical relationships found in things with fuzzy characteristics. A comprehensive fuzzy evaluation is an important application of fuzzy mathematics. When the circumstances involve very complex factors, it can be used for selecting the best program for execution or making a choice after ranking the system detection results after the evaluation [36–38].

The main steps of the fuzzy evaluation method are as follows: (i) determine the factors and comments sets for evaluation, and then establish the fuzzy sets of the various factors (membership function); (ii) establish the fuzzy relationship between the evaluation factors and the comments, and then determine the weight that the respective factors have during evaluation; and (iii) derive a conclusion on the basis of calculations using a specific operand. Flexibility in the handling of attacks and the use of reasonable judgment are required for identifying a strict boundary between the normal and the abnormal [39, 40].

We have used the fuzzy sets technique in this study. The fuzzy sets of basic variables are represented by the following quintuple:

(1) $$\begin{array}{c} \text{Fuzzyset}::=\langle \text{Object},\text{Attribute},\text{FC},\text{Domain},\text{ML}\rangle . \end{array}$$

Here, Object refers to the item being described; Attribute, a particular property of the object; FC, the fuzzy concept; Domain, the location of the attribute; and ML, the membership list.

The procedure for conducting a fuzzy evaluation is as follows [41, 42].

**Step 1**

Determine the factors and comments sets for evaluation, and then establish the fuzzy sets of the various factors. Internet access can be described using various characteristics such as the duration of the connection, communication volume, source and destination addresses, and types of service (i.e., the target port number). A compilation of these characteristics is known as the factors set. The evaluation vector is the bituple *E* = 〈*U*, *W*〉, where *U* denotes the factors set *U* = {*u*_1_,  *u*_2_,…, *u*_*n*_} and *W* represents the weight vector. Every component of *W* corresponds to the degree of importance of a factor during evaluation and can be represented as follows: *W* = ∫_*u*_ *w*/*u*. Corresponding to the factors set is the comments set, which refers to the set of linguistic variables of the condition “degree of abnormality.” The method of describing each factor is consistent. Therefore, the density distribution function of these factors can be treated as their membership function. During this step, the task is to calculate the density distribution function of each factor using the existing data.

**Step 2**

**Figure 2:**
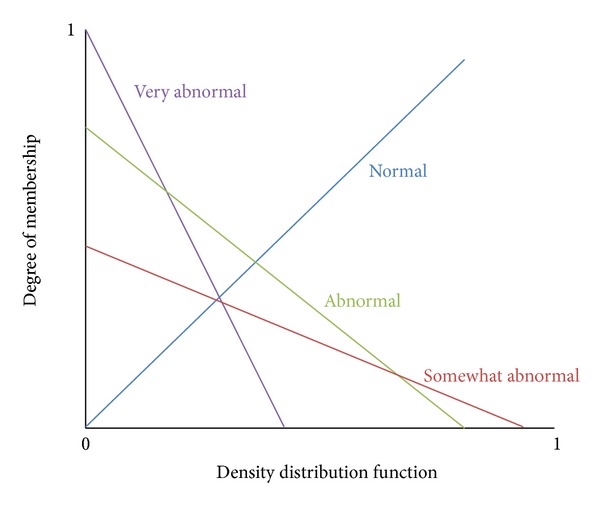
Membership function of the various comments.

Evaluate the fuzzy relation between the factors and comments sets, and then determine the weight to be ascribed to the various factors during evaluation. This is the most important step in intrusion detection based on fuzzy evaluation. The detection model can be established once the fuzzy relation between the two sets has been determined. The fuzzy relation between the factors *u*_*i*_ and comments indicates the degree of membership that the respective factors have with the various degrees of abnormality. The determination of the fuzzy relation between the factors *u*_*i*_ and comments *e*_*j*_ is based on *f*(*u*_*i*_), which is the density distribution function of *u*_*i*_. If the comments set is {*e*_1_,  *e*_2_,…,  *e*_*m*_}, then the density distribution function of *u*_*i*_ will be mapped onto *m* number of fuzzy relations. The relationship between the membership functions of a fuzzy relation is shown in Figure 2. The following two characteristics of the fuzzy relation between the factors and the comments can be identified from Figure 2.

- The smaller the density of a particular eigenvalue is, the greater the degree of membership of the comment is to a higher degree of abnormality.
- The higher the degree of abnormality of a comment is, the larger the membership function slope is.

In order to determine the weight of each factor, it is necessary to assess and rank the importance of all the factors. In this study, a judgment matrix established through the expert evaluation method (EEM) was used. The EEM is an important fuzzy mathematics tool used for creating fuzzy sets, fuzzy relations, and other mathematical models. It relies mainly on the experience of experts in the related fields. The sequence to establish a judgment matrix using EEM is as follows.

- Invite *n* number of experts to establish a comparative judgment matrix *A*_1_, *A*_2_,…, *A*_*n*_ for a particular type of intrusion, on the basis of their own experiences and the concept of fuzzy relations.
- Set up a group of weights *W*_1_, *W*_2_,…, *W*_*n*_,  *W*_1_ + *W*_2_ + ⋯*W*_*n*_ = 1 in accordance with the authority ranking of the experts, where *W*_*i*_ represents the authority ranking of expert number *i*,  *i* ∈ 1,  2,…, *n*.
- Represent the final judgment matrix as *A* = *W*_1_ × *A*_1_ + ⋯+*W*_*n*_ × *A*_*n*_.

**Step 3**

The conclusion from the evaluation and calculations carried out using a particular operand is derived as follows.

- Use the comments set to assess each eigenvalue that was determined by the aforementioned fuzzy relations, and then compose the evaluation matrix.
- Carry out a compositional operation of the fuzzy matrix using the weight vector of the factors list and the evaluation matrix, thereby deriving a comprehensive evaluation vector.
- Determine the comments for this particular set of eigenvalues on the basis of the principle of the maximum degree of membership.

### 4.4. Proactive and Early Security Warning Mechanism

**Figure 3:**
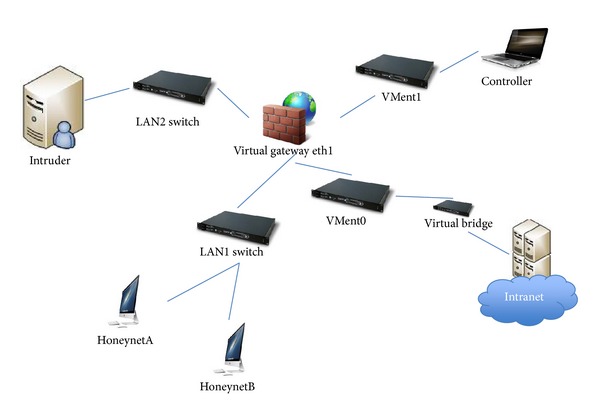
Membership function of the various comments.

The warning mechanism used in this study is a structurally implemented network based on a closed-ended virtual honeynet. Except for the managing platform, the virtual honeynet does not carry out any interactive data transmission with any external host or device. The closed-ended virtual honeynet comprises a virtual intruder, a virtual honeynet gateway, and two virtual honeypot systems. Its network topology is shown in Figure 3.

Within the virtual honeynet, the LANl, LAN2, VMnet0, and VMnetl switches are all virtual Layer 2 switching equipment. The LANl and LAN2 switches control the exchange of data between the virtual devices. The VMnet0 and VMnetl switches exercise similar controls but between the virtual devices and the host (managing platform). The VMnetl switch uses the host mode to ensure that the homed host (managing platform) can manage the honeynet gateway; that is, information from the homed host can be transferred to Interface Number 2 (eth2) of the honeynet gateway. On the other hand, the VMnet0 switch uses the bridging mode (normally not used in a closed-ended virtual honeynet) to ensure that the data pass directly through the physical network interface of the homed host (managing platform) to the real network.

There is a bridge between Interface Numbers 0 (eth0) and 1 (ethl); hence, these do not have any IP address. When data packets pass through the gateway, their time-to-live (TTL) values are not reduced. As such, the honeynet gateway is not visible to virtual intruders. A virtual honeynet system can provide system administrators with the ability to monitor, defend, and document the security of all segments of the network and can play a significant role in enhancing the security management of network systems.

Further, data control is also a very important concept. The main purpose of data control is to prevent intruders from using the honeynet as a springboard to send illegal information outside or attack other machines, after having obtained the administrative rights to the honeypot. Whenever intruders initiate any actions to scan, probe, or connect to the honeynet from the outside, these items must be captured. All scans, probes, and connections being made from the honeynet to the outside must also be strictly controlled and released subject to conditions. Data packets found with abnormalities must be blocked. At the same time, intruders must not realize that their behaviors are being monitored.

The main tool used for achieving the objective of data control at the honeynet gateway is the hybrid firewall, whose design was mentioned earlier. The firewall limits the frequency of outgoing connections. Since the data packets that must be controlled are being forwarded, the *m*-limit feature of Iptables can be used for implementing this function. The unit of time for counting purposes can also be specified, for example, by the seconds, minutes, or hours. If the number of outgoing data packets reaches the upper limit within a designated time period, the feature will record the relevant information and alert the system administrators accordingly. The data packets to be monitored include various types of information. In addition to the Transmission Control Protocol (TCP), User Datagram Protocol (UDP), and Internet Control Messaging Protocol (ICMP) packets, other data packets with unknown protocols must also be monitored. Further, specific rules can be set using the rc.firewall script. The actions to take against suspicious data packets include deletion, prohibition, or disposal.

### 4.5. Blocks Extensible Exchange Protocol (BEEP)

We introduced the BEEP [43] concept and conducted a more in-depth study of its applications [44, 45] while implementing connectivity and interactivity between the firewall system, IDS, and the virtual honeynet system. BEEP concretizes the concept of messages into useful communication units. These messages, which form a part of the application protocol conversation, can be “The temperature of my CPU is 70°,” or “This is a JPEG image.” Since MIME is used as an envelope, these messages can be in any form or type. There is no actual limit on the size of the messages either. Rather, the message size is determined by the specific applications involved in the transmission. Therefore, BEEP is implemented by frames, communication units that are smaller than the messages. Although a message can often be reasonably sent out via one frame, it can also be split into multiple frames when necessary. A frame contains information that identifies the channel to which it belongs, the header of the message, and its sequential order within the entire message.

The existing implementation plans for the BEEP framework include Beepcore-c, Beepcore-j, and RoadRunner. Beepcore-c was adopted as the implementation method of BEEP in this study. The features of Beepcore-c are as follows.

- BEEP is implemented using C/C++.
- It can achieve NULL/ECHO, NULLSINK, SASL/Anonymous, SASIJOTP, and TLS profile.
- It has a hierarchical structure. The software structure is divided into four layers from top to bottom: Core, Wrapper, Profile Implementations, and Application.

Next, we used the OpenSSL library [46] to implement SSL and TLS. OpenSSL has the following characteristics.

- It is open source.
- It supports SSL v2/v3 and TLS v1.
- It is stable and supports the SSL and TLS properties completely.

After OpenSSL is fully compiled, two dynamic link library (DLL) files are generated: libeay32.dll and ssleay32.dll. The TLS profile of Beepcore-c contains the commands to call out these dynamic link library files.

Next is the implementation of the BEEP protocol over TCP. RFC3081 defines the implementation method to map BEEP onto the TCP protocol. Even though TCP provides the flow control for each session, a BEEP session may contain multiple simultaneous BEEP channels. As such, BEEP must provide a solution to avoid deadlocks. Hence, when BEEP is introduced to the TCP flow management mechanism, each channel's window size must be dynamic. Window sizes are exchanged between peers through SEQ.

The next issue to address is that of SMIP configuration. Under the pcore-c framework, profile realization is based on the DLL format. DLL calls out the pro init function, which is used by the SMIP Listener and Initiator to complete profile registration. During the registration process, in order to take into account the PROFILE_REGISTRATION structure, URI, Initiator_modes, listener_modes, the other values must be initialized and be sent to the corresponding callback function at the same time (Wrapper will call back these functions in due course).

The SMIP profile carries out its functions and roles via two aspects: Listener and Initiator. The former implements the monitoring function. When it has completed profile registration and receives a peer connection request from BEEP, Wrapper first uses pro_connection_init to complete profile initialization. Next, it uses the pro_session_init function to complete the initialization of the BEEP conversation. Thereafter, bpc start response is used for completing the establishment of the BEEP channel. SMIP will then go into the monitoring mode. On the other hand, after Initiator completes initialization using pro_connection_init and pro_session_init, it uses bp-start request to complete the connection request. At this time, profile negotiation between the peers is completed and MSG is sent to exchange SMIP-greetings. After the completion of profile negotiation, a data exchange can be carried out between the peers through the SMIP profile. Depending on the options in the configuration file, the SMIP profile can complete user authentication by using either TLS, SASL/Anonymous, or SASI/OTP profile. TLS then completes the encryption process for EMEF message transmission.

Now, we will discuss the issue regarding IMEF data format. In the program, a Clmefmessage class is used for completing the packaging of the IMEF message. Within the ClmefMessage class, an IMEF message structure is defined and used for storing the original unformatted data of the IMEF message. The IMEF message structure is converted by the CIamefMessage::MakeXMLImefMessageQ member function into an XML format that complies with the LMEF XML DTD definition to facilitate the connecting and interacting functions.

The managing components for connectivity and interactivity include the event engine and analysis modules, as well as the strategic module for connectivity and interactivity. The details are as follows.

- The event engine module is supported by the BEEP protocol. It is used for facilitating communication between the firewall and other security systems, as well as for receiving event information sent by various types of security systems. It then adds the received event information to the predefined events queue.
- The event analysis module extracts events from the events queue and matches these against the library of strategies for connectivity and interactivity. Depending on the category of security event identified, it then selects the appropriate response strategy.
- The strategic module for connectivity and interactivity is mainly used for effectively responding to the various intrusion behaviors existing in the actual network environment. This module can be used for the configuration and management of the entire library of strategies, thus ensuring that connective and interactive responses are carried out smoothly.

**Figure 4:**
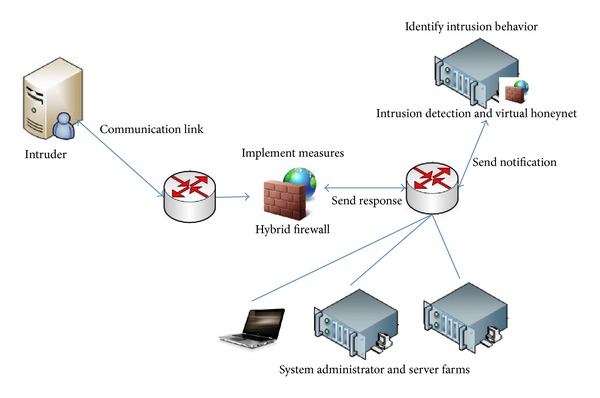
Schematic representation of a network intrusion.

We would like to illustrate the process with a typical case of network intrusion (Figure 4). After the IDS detects an intrusion in the network or host by a hacker, it communicates with the firewall and the virtual honeynet interface through the BEEP protocol to lure the network intruder into the trap host. It then analyzes the characteristics of the intruder and notifies the firewall to generate dynamic rules to control and block the intrusion. Depending on the nature of the intrusion and the level of risk, the firewall can produce many different dynamic rules, each with a specific time period of effectiveness. This facilitates a variety of controlled operations, including early warning, termination of current conversation, and blocking of all connections from a particular source. After the firewall has implemented the measures, it reports the results to the system administrators and generates a log file.

## 5. System Implementation Test

Out of safety considerations, we only carried out tests targeting the laboratory's internal setup. Some common hacking tools were used for simulating an intrusion of the entire model. Two detection engines were installed prior to system intrusion. One was a network engine that received data packets from the intranet, and the other was a host engine that received data packets from the database server. Both engines collected the intrusions that each had respectively analyzed and then sent the information to the system console located in the intranet.

The operating system for the system console was Windows 2008 Server. The firewall and the virtual honeynet system used Linux, and the database used MySQL. The two machines that initiated the intrusion were located outside the network. The IP address of Attacking Hosts 1 and 2 was 122.116.76.141 and 122.116.76.147, respectively. The targets of the attack were Hosts 1 and 2 and the database server of the 192.168.0.0 subnet. The IP address of Hosts 1 and 2 was 192.168.0.9 and 192.168.0.11, respectively. The IP address of the database server was 192.168.1.10 (Windows 2008 Server was the operating system installed).

### 5.1. Tests for DoS Attacks

The log information of the hybrid firewall indicated that the attacker initiated the following connection:

 2013-03-06, 09:48:05, 192.168.1.10, 2, Blocking host 122.116.76.147 completely for 600 seconds
 2013-03-06, 09:48:05, -, l, iptables, Info: Blocking ip 122.116.76.147.

The data captured by Snort indicated that this was a typical DoS test attack. The records in the system log files were as follows:

 2013-03-06 09:48 snort[1852]: [1 : 474 : 1] Dos [Classification: Attempted Information Leak] [Priority: 2]:: Dos 192.168.0.10
 2013-03-16 09:48 in.rlogind[1316]: connect from 192.168.0.10
 2013-03-16 09:48 inetd[413]: pid 1318: exit status 1
 2013-03-16 09:48 in.rshd[1318]: connect from 192.168.0.10
 2013-03-16 09:48 in.fingerd[1315]: connect from 192.168.0.10
 2013-03-16 09:48 in.telnetd[1313]: connect from 192.168.0.10
 2013-03-16 09:48 rshd[1318]: Connection from 192.168.0.10 on illegal port
 2013-03-16 09:48 telnetd[1313]: ttloop: peer died: EOF
 2013-03-16 09:48 inetd[413]: pid 1316: exit status 1
 2013-03-16 09:48 inetd[413]: pid 1313: exit status 1
 2013-03-16 09:48 sendmail[1314]: NOQUEUE: Null connection from [192.192.0.10]
 2013-03-16 09:48 in.fingerd[1319]: connect from 192.168.0.10
 2013-03-16 09:48 in.telnetd[1320]: connect from 192.168.0.10.

In response, measures were taken by the firewall to block the attack. The records in the system log were as follows:

 2013-03-16, 09:48 : 10, -, l, iptables, Info: Command/sbin/iptables -I FORWARD -i eth0 -s 122.116.76.147 -j REJECT Executed Successfully
 The list of rules generated by the firewall included the following:
 Chain INPUT (policy ACCEPT)
 target port opt source destination
 Chain FORWARD (policy ACCEPT)
 target port opt source destination
 REJECT all –122.116.76.147 anywhere reject-with-icmp-port-unreachable
 Chain OUTPUT (policy ACCEPT)
 target port opt source destination.

The above records showed that the DoS attack launched by the IP address 122.116.76.147 was promptly arrested by the firewall. The measure adopted in response was to block this IP address.

### 5.2. Tests for Different Types of Intrusion

The types of network intrusion used for the experiment are shown in Table 1.

Set the features set as *F*, where *F* = {*f*_1_, *f*_2_,…, *f*_*n*_}, and the comments set as *E*, where *E* = {*e*_1_, *e*_2_,…, *e*_*m*_}. Assess the individual factors to obtain the evaluation vector *R*_*i*_ = (*r*_*ij*_),  *i* = 1,2,…, *n*,  *j* = 1,2,…, *m*, followed by the evaluation matrix *R* ^*T*^ = [*R*_1_,  *R*_2_,…, *R*_*n*_]. The fuzzy comprehensive evaluation matrix (with the weight of the features being set to *W*_*E*_ = (*W*_*f*_1__, *W*_*f*_2__,…, *W*_*f*_*n*__)) can then be obtained via fuzzy composition as follows:

(2) S=(S1,S2,…,Sn)=(Wf1,Wf2,…,Wfn)×[r11r12⋯r1mr21r22⋯r2m⋮⋮⋮⋮rn1rn2⋯rnm].

Set *S*_*K*_0__ = max(*S*_*j*_), *i* = 1,2,…, *n*, so that this particular factors set belongs to level *K*_0_. The intrusion event is a subset of the comments set. The comments set created for the experiment comprises four comments, namely, “Normal,” “Somewhat abnormal,” “Abnormal,” and “Very abnormal”.

All comments assessed as “Abnormal” and above are classified as intrusions. At the start of the experiment, the tcp dump within the gateway was activated to collect the network data. The outputs of this process were multiple records of network communication. These records were divided into four groups. Group 1 was the baseline, which contained network data that did not relate to intrusion activities. In this group, 80% of the data were treated as training data, while the remaining 20% were used for testing the misreported rate. The data for Groups 2–4 were contained in network1, network2, and network3, respectively. Each group was subjected to three different types of attacks. After processing, the four groups of data used for testing the detection rates were stored in the database. The names of the network connection tables were “Normal,” “Intrusion1,” “Intrusion2,” and “Intrusion3,” respectively.

During the experiment, network connections were divided into three categories: (i) outgoing network connections from the local network; (ii) incoming network connections from the extranet; and (iii) connections within the local area network (LAN). The results of the experiment are shown in Table 2.

Selective data were used for testing. The statistics related to the intrusion detection subsystem are shown in Tables 3 and 4. The former is based on the information collected and the latter on the intrusion type.

**Figure 5:**
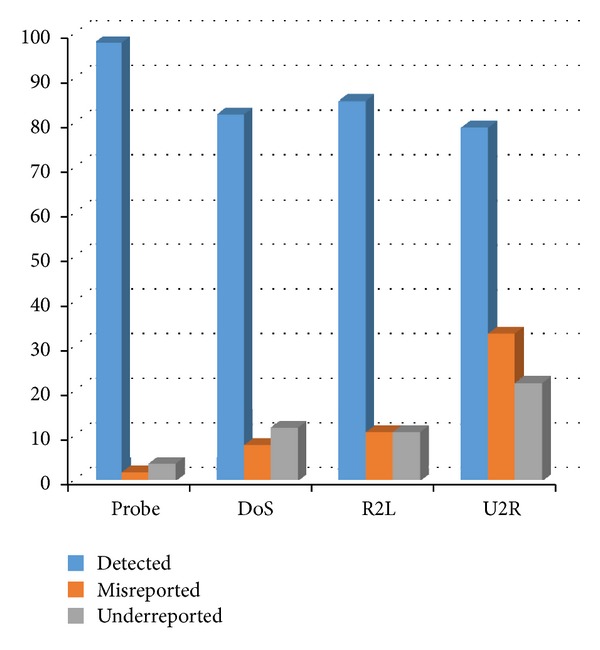
System performance indicators.

The system performance indicators of the network security early warning system were derived through further calculations and analysis. The data are shown in Table 5, and the graphical representation is presented in Figure 5.

Computed using the above data, successful detection rate was 87.15%. The requirements that we set for the experiment were met to a certain extent. The firewall system and IDS were stable in their operations and fully functional. The analysis of the two sets of experimental data indicated that the security protection of the system was further enhanced through the connectivity and interactivity between the firewall, IDS, and virtual honeynet. As long as the proposed network security architecture study initiated a connection, the system responded in a timely manner, irrespective of the mode of attack. The connection was also recorded and documented in the system log. This provided a comparative analysis to the system administrators and enabled them to take appropriate measures promptly.

With the above notwithstanding, the experiment reflected a number of shortcomings:

- although the IPSec protocol of the firewall could protect the security of the data packets, it reduced their transmission speeds;
- there was room for further strengthening of the system's self-adaptability.

## 6. Conclusion

In this study, we used an actual implementation to validate the performance of the designed network defense model. The experimental data indicated that the entire system had an average successful detection rate of 87.15%, which met the design requirements. However, a certain margin of error still existed. Further, the firewall system, IDS, and virtual honeynet system had stable operations, were fully functional, and could fulfill the design requirements.

The contributions of this study could be summarized as follows.

- The concept of a network defense model was proposed shortly after a systems analysis of a sensitive and classified network was carried out. This systems analysis was an important prerequisite of and the basis for systems design. In this study, a model for an intelligent early warning system was designed. Based on the systems methodology and a combination of the theories of network security and the principles of automatic control, the proposed model was self-adaptive and could respond to network security issues in a dynamic manner.
- IPS technology was used for establishing a distributed network security architecture comprising the following components.
  - Hybrid firewall: A hybrid firewall system was designed on the basis of packet filtering and proxy and VPN technologies.
  - IDS: Snort tools were used for creating a network intrusion detection system that used a fuzzy comprehensive evaluation to determine the intrusion detection eigenvalues.
  - Virtual honeynet system: The approach of a virtual network trap was adopted, together with the implementation of a close-ended virtual honeynet, to give a proactive and early security warning to the network.
  - Connectivity and interactivity were established between the firewall, intrusion detection, and virtual honeynet, which further proved the practicality and usability of the system.

Distributed network security architecture [47–49] can effectively prevent network intrusions and provide direct protection for key data. It plays an important supporting role in the construction of a network security system. Not only can it be applied to the development of a academic network, but it can also be used for constructing and improving the networks of private corporations and governmental network. The results of this study can provide new ideas and solutions, as well as serve as a reference for future network security topology design and related studies.

## 7. Future Research Direction

Given the multiple topics under the umbrella of network security, the following four aspects in this study were focused on: firewall, intrusion detection, virtual honeynet, and connectivity and interactivity between these three components. After a long period of research and actual implementation and on the basis of an extensive analysis, we could successfully establish and verify the entire architecture. However, because of the limitations of various factors, including knowledge, team experience, time, and individuals' abilities, we could not completely resolve all the issues related to the network security architecture.

The issues that require further research include the following:

- intrusion detection technologies: to expand the scope of research by using this study as the basis and to continuously improve and refine data detection technologies in order to improve data detection efficiency,
- distributed security communications: to strengthen related research such that data transfers between security components can be safer, more effective, and immediate,
- data security: to carry out further research on encryption and transmission,
- information on early warnings, storage of such information, and the operating mode of the information database: To conduct appropriate research in the related areas.

## Figures and Tables

**Algorithm 1:**
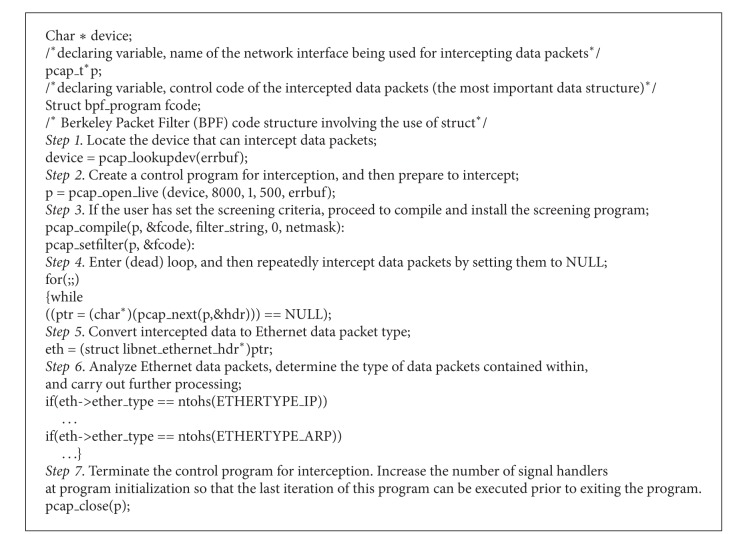


**Algorithm 2:**
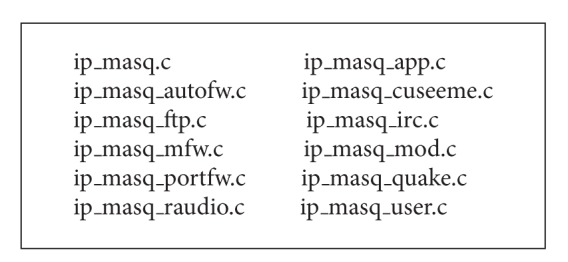


**Algorithm 3:**
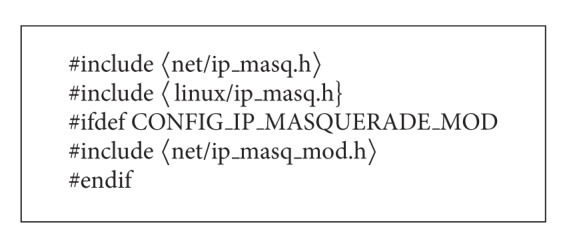


**Table 1:** Types of intrusion.

Category	Type	Activities
0	Normal	Normal
1	Probe	Probes on system vulnerabilities, for example, port scans
2	DoS (Denial-of-Service)	DoS attacks, for example, SYN flooding
3	R2L (Remote-to-Local)	Unauthorized access by remote machine, for example, password guessing
4	U2R (User-to-Root)	Unauthorized access by locally managed accounts, for example, buffer overflow attacks

**Table 2:** Intrusion ratio of normal and abnormal data.

Network connection table	Ratio of network connections evaluated as intrusions		
	Outgoing connections	Incoming connections	Connections within the LAN
Normal	0.62	0.23	0.88
Intrusion 1	3.09	15.44	18.96
Intrusion 2	3.81	12.83	12.42
Intrusion 3	2.41	20.03	9.51

**Table 3:** Statistics based on information collected.

Data	Number of cases			
	Intrusion	Misreported	Underreported	Detection rate (%)
Dataset 1	94	13	8	92%
Dataset 2	90	8	10	89%
Dataset 3	88	7	8	91%
Dataset 4	96	12	5	95%

**Table 4:** Statistics based on intrusion type.

Type	Number of cases			
	Detected	Misreported	Under-reported	Total
Probe	226	4	7	233
DoS	131	14	23	155
R2L	77	12	12	89
U2R	29	12	8	36

**Table 5:** System performance indicators.

Performance indicator %	Type of attack				
	Probe	DoS	R2L	U2R	Mean
Detected	96.99	84.51	86.52	80.56	87.15
Misreported	1.71	9.03	13.48	33.33	14.39
Under-reported	3.01	14.84	13.48	22.22	12.36
